# Satisfaction With Internet Access, Cancer Information-Seeking, and Digital Health Technology: Cross-Sectional Survey Assessment

**DOI:** 10.2196/69606

**Published:** 2025-08-27

**Authors:** Maria Andrea Rincon, Richard P Moser, Kelly D Blake

**Affiliations:** 1 Division of Cancer Control and Population Sciences, National Cancer Institute, 9000 Rockville Pike, Bethesda, MD, 20892, United States, 1 2400553564

**Keywords:** digital health, cancer, health information-seeking, telehealth, EHR, electronic health record

## Abstract

**Background:**

Access to high-quality internet plays an increasingly important role in supporting care delivery and health information access. Although internet access has the potential to alleviate some inequities in health care, the digital divide negatively impacts cancer across the continuum. While subscription to high-speed internet has been previously assessed, satisfaction with home internet to meet the health needs of users is a lesser-known, important indicator of satisfactory access to internet-based health information and digital health technology use.

**Objective:**

This study aimed to assess differences in perceptions of quality of at-home internet connection and its association to cancer health information-seeking experiences and use of digital health technologies in a nationally representative sample of US adults.

**Methods:**

Secondary analysis of data from the National Cancer Institute’s Health Information National Trends Survey (HINTS) 2022 (n=6252) was conducted. The primary predictor, “how satisfied are you with your Internet connection at home to meet health-related needs?,” a novel item on HINTS 6, was dichotomized into “high” (extremely satisfied or very satisfied) and “low” (somewhat satisfied, not very satisfied, or not at all satisfied) satisfaction. Outcomes variables included 3 items assessing cancer information-seeking experiences and 2 items measuring access to telehealth and patient portals over the past 12 months. Adjusted logistic regression models (*P*<.05) were performed, including age, race and ethnicity, education, income, health insurance access, geography, and difficulty understanding cancer information, a proxy for health literacy, as covariates.

**Results:**

Those reporting low satisfaction with their home internet had higher odds of agreeing that searching for cancer information took a lot of effort (odds ratio [OR] 1.59, 95% CI 1.16‐2.19) and that they felt frustrated searching for cancer information (OR 1.46, 95% CI 1.07‐1.98). Respondents with lower satisfaction with their home internet had lower odds of accessing their patient portal at least once in the past year (OR 0.54, 95% CI 0.33‐0.89). While the relationship between internet satisfaction and concern over information quality was not significant, respondents aged 18‐34 years reported higher odds to be concerned compared with those aged 75 years and older (OR 1.74, 95% CI 1.04‐2.90), and those with lower education reported less concern over the quality of information compared with those with postbaccalaureate degrees (high school graduate: OR 0.56, 95% CI 0.31‐0.99; college graduate: OR 0.67, 95% CI 0.48‐0.95). Finally, while the association between satisfaction with internet and telehealth use over the past 12 months was not significant, those without health insurance were significantly less likely to have had a telehealth appointment in the last year (OR 0.39, 95% CI 0.19‐0.81).

**Conclusions:**

Satisfaction with internet at home to meet health needs is correlated with cancer information–seeking experiences and usage of some available health technology. These findings underscore the value of high-quality internet services toward successful implementation of health care technology and better patient experiences in health information seeking.

## Introduction

### Background

High-speed internet, typically known as broadband internet, which is measured by established upload and download speeds, allows users to access needed information and services with greater efficiency compared with dial-up services [1]. Access to high-speed internet plays such a critical role in our daily lives that it has been deemed a “super determinant of health” due to its direct impact on other social determinants of health; it can also influence health behaviors, such as using telemedicine to connect specialists to patients in remote, underserved locations [2-4]. Understanding the importance of broadband access, financial incentives such as the Centers for Medicare and Medicaid Services EHR (electronic health record) Incentive Program, also known as Meaningful Use Program, were created to support widespread implementation of internet-driven resources throughout health systems [1,5-7]. These systems were tested by the COVID-19 pandemic which placed an unprecedented burden across health care providers and systems worldwide, leading to reliance on health information technology resources, including a rise in the use of telehealth to provide care for those seeking care or infected, and sustain care for chronically ill patients, including those with cancer [8-10].

Studies examining the digital divide suggest that lack of access to broadband internet poses a significant barrier to access, implementation, and ease of using digital health technologies. A recent study assessing disparities in access to electronic health records using data from the American Community Survey found respondents from neighborhoods where less than 85% of the neighborhood had broadband access were significantly less likely to access their EHR [11]. Furthermore, assessment of broadband capacities has found that areas with the highest broadband availability have significantly greater use of telehealth services, compared with the neighborhoods with lower broadband access [12,13].

Lack of access to internet-based information is especially problematic for cancer survivors who have unique information needs. A 2023 study using a 17-item Digital Inequity Index on esophageal cancer outcomes found increasing digital inequity, both infrastructural and sociodemographic, was associated with decreased survival and long-term follow-up, lower odds of receiving chemotherapy, and higher odds of late-stage diagnosis [14]. High-speed internet connectivity is particularly relevant to remote areas that experience high cancer rates, such as rural Appalachia [15-17]. Unequal access to care among rural cancer patients has resulted in consistently worse outcomes compared with their urban counterparts, even as diagnostics and treatment modalities improve [18-20]. While the incorporation of telehealth services and use of digital health technology has been proposed to reduce such disparities, lack of broadband internet in many of these regions may hinder the potential of these interventions. A study on the relationship between broadband access and use of telemedicine found that in counties classified as “fully rural” (ie, nonmetropolitan counties without urban areas, based on the United States Department of Agriculture (USDA) Rural-Urban Continuum Codes), broadband availability was associated with access to telemedicine, with counties with low broadband access showing 34% fewer visits per capita compared with counties with greater broadband access [21]. This reduction was also seen in the results of a cross-sectional survey that showed that cancer patients who lacked at-home broadband access were likely to report lower scores related to their comfort with technology [22].

Given the importance of broadband internet to health, understanding who has access and who does not is critical. However, most people are not aware of their internet upload and download speeds, and so their perceptions of the quality of their internet may be a more important indicator of access [23,24]. Quality of internet connection can pose significant barriers to implementation of remote health services. A retrospective analysis of telehealth services in Wisconsin homes during the COVID-19 pandemic showed that more than 15% of telehealth appointments with poor internet quality failed to connect, and over 1 in 20 encounters overall were converted into telephone-only appointments [25].

### Objectives

The objective of the current study was to assess disparities in perceptions of the quality of at-home internet connection to meet health needs, using a novel item addressing respondent satisfaction with at-home internet at a population level in a representative sample of US adults. Our primary aims were to determine differences in self-reported satisfaction with at-home internet connection by sociodemographic characteristics, including age, educational attainment, race and ethnicity, household income, health insurance, rural or urban household designation, and difficulty understanding cancer information, and to document the association between internet satisfaction and cancer health information-seeking experiences and use of digital health technologies in this population.

## Methods

### Data Source

Data from the National Cancer Institute’s Health Information National Trends Survey (HINTS), collected between March and November 2022, were used (HINTS 6). Multimedia Appendix 1 provides a description of the dataset, using the Preferred Reporting Items for Complex Sample Survey Analysis (PRICSSA), an itemized checklist that provides guidance to researchers on reporting the use of complex sample survey data for research studies [26].

### Predictors and Outcomes

Our primary predictor variable, satisfaction with at-home internet connection, was measured using one item from HINTS 6: “How satisfied are you with your Internet connection at home to meet health-related needs?,” with a 5-point scale response, ranging from “extremely satisfied” to “not at all satisfied.” For brevity purposes, we will refer to our predictor variable as “satisfaction with at-home Internet” in mentions to follow. In the analysis, responses were reverse coded and dichotomized into “high satisfaction” (extremely satisfied or very satisfied) and “low satisfaction” (somewhat satisfied, not very satisfied, or not at all satisfied). For the outcomes of cancer information-seeking experiences, 3 items were included: “Based on the results of your most recent search for information about cancer, how much do you agree or disagree with each of the following statements?:” (1) “It took a lot of effort to get the information you needed;” (2) “You felt frustrated during your search for the information;” and (3) “You were concerned about the quality of the information.” Each of these items included a 4-scale response, ranging from “strongly agree” to “strongly disagree.” Items were recoded as dichotomous variables, with the agreement category including “strongly agree” and “somewhat agree” and the disagreement category including “strongly disagree” and “somewhat disagree” responses. Responses to the 3 items described above were limited to participants who replied “yes” to a previous question: “Have you ever looked for information about cancer from any source?”

To assess the association of satisfaction with at-home internet and use of health information technology, 2 items from HINTS 6 were used. To determine use of telemedicine, we created a dichotomous (Yes or No) outcome based on the item that read: “In the past 12 months, did you receive care from a doctor or health professional using telehealth?.” Valid, affirmative response options (“yes, by video”; and “yes, some by video and some by phone call”) were collapsed into a single category. Those who reported “no telehealth visits in the past 12 months” or “yes, by phone call [voice only no video]” composed the “No” category. For this outcome, responses were limited using a filter variable (“In the past 12 mo, not counting times you went to an emergency room, how many times did you go to a doctor, nurse, or other health professional to get care for yourself?”); in this case, only respondents who reported 1 or more visits to a health care professional in the past year were included as part of the analysis. Access to electronic health records or patient portals was examined through the item that read: “How many times did you access your online medical record or patient portal in the last 12 months?” Response categories with valid answers (“0”, “1 to 2 times,” “3 to 5 times,” “6 to 9 times,” or “10 or more times”) were dichotomized into 2 categories: 0 and 1 or more times. Respondents who reported not having an electronic medical record or not having been offered one by a provider or insurer, and those with missing data, were excluded from the analysis.

All models in our analysis were adjusted for the following covariates: age (18‐34 years, 35‐49 years, 50‐64 years, 65‐74 years, 75 years and older); highest level of educational attainment (less than high school, high school graduate or GED, some college or vocational education, college graduate, postbaccalaureate degree); annual household income from all sources (less than US $20,000, US $20,000‐34,999, US $35,000‐49,999, US $50,000‐74,999, US $75,000‐99,999, US $100,000‐199,999, and US $200,000 or more); household geographic location, collapsing the 9 designations from 2013 USDA into 2 categories (urban or rural); and difficulty understanding cancer information (“Based on the results of your most recent search for information about cancer, how much do you agree or disagree with each of the following statements? The information you found was hard to understand”), which was dichotomized (strongly agree or agree and strongly disagree or disagree) and used as a proxy to control for health literacy in our analysis. Reference categories for each covariate were selected to show potential disparities for less fortunate groups compared with groups with more access to resources, such as those with highest educational attainment, living in urban areas, with access to health care insurance and reporting a household income within the highest category.

### Analysis

Due to the complex sampling structure of HINTS 6, all analyses incorporated a final sample weight for population-level point estimates, and 50 replicate weights to calculate accurate standard errors, where the latter used the Jackknife minus one replication method. To assess the bivariate association between satisfaction with home internet and sociodemographic characteristics of the HINTS respondents, Rao-Scott chi-square tests were used. These sociodemographic variables were also included in the adjusted models: age, educational attainment, race and ethnicity, annual household income, access to health insurance, USDA’s 2013 urban or rural designation, and difficulty understanding cancer information, given their theoretical and statistical relationships with the outcomes. Differences across sample sizes in the analytical models are present due to skip patterns and missing data in variables included within each model.

Because dependent variables of interest were dichotomized, we used multivariable logistic regression models to determine the association between satisfaction with at-home internet connection and each of our outcomes: (1) effort getting information needed (n=2390); (2) frustration during search for information (n=2386); (3) concern about the quality of information (n=2391); (4) accessing online medical record or patient portal in the last 12 months (n=2179); and (5) receiving care from a doctor or health professional using telehealth in the last 12 months (n=2212). All statistical analyses were conducted using SAS (version 9.4; SAS Institute) using the complex survey-related procedures.

### Ethical Considerations

The HINTS 6 general population survey was designated “exempt research” under 45 CFR 46.104 and approved by the Westat Institutional Review Board (IRB) on May 10, 2021 (project #6632.03.51), with a subsequent amendment approved on November 24, 2021 (amendment ID #3597). HINTS 6 also received a “Not Human Subjects Research” determination from the National Institutes of Health Office of IRB Operations on August 16, 2021 (iRIS reference number: 562715). This study is a secondary analysis of HINTS 6, a publicly available, deidentified dataset. In accordance with US federal regulations (45 CFR 46) and institutional policies, secondary analyses of deidentified, publicly available data do not require additional IRB review. Further details regarding the primary data collection procedures, including informed consent and participant compensation, can be found in the HINTS 6 methodology report [27].

## Results

### Sociodemographic Sample Characteristics

Sociodemographic predictors, stratified by high and low satisfaction with home internet to meet their health needs, are presented in Table 1. This table includes unweighted values for all characteristics, as well as weighted percentages in each of the categories included. Of the categories presented, educational attainment, ease in understanding cancer information, household income, and geographic region were all significantly associated with the high and low satisfaction with internet categories (*P*<.05).

**Table 1.** Sociodemographic characteristics (unweighted sample size and weighted percentages) of Health Information National Trends Survey (HINTS) 6 respondents, by high versus low satisfaction with home internet to meet health needs.

Satisfaction with home internet to meet health needs	High, n (%)	Low, n (%)	All, n (%)	*P* value
Age (years)				<.001
18‐34	652 (20.2)	222 (7.6)	874 (27.8)	
35‐49	823 (19.3)	309 (7.7)	1132 (27)	
50‐64	985 (18.4)	522 (9)	1507 (27.4)	
65‐74	641 (7.1)	392 (4.5)	1033 (11.6)	
75+	273 (3.2)	247 (3)	520 (6.2)	
Education level				<.001
Less than high school	99 (3)	76 (1.6)	175 (4.6)	
High school graduate	380 (11.8)	300 (6.9)	680 (18.7)	
Some college	858 (25.8)	540 (14.8)	1398 (40.6)	
Bachelor’s degree	1045 (14.8)	432 (5.8)	1477 (20.6)	
Postbaccalaureate degree	794 (12.3)	255 (3.2)	1049 (15.5)	
Understanding cancer information^a^				.002
Easy to understand	1239 (46.8)	458 (16.8)	1697 (63.6)	
Hard to understand	572 (23)	334 (13.4)	906 (36.4)	
Race and ethnicity				
Non-Hispanic White	1904 (43.8)	896 (19.5)	2800 (63.3)	
Non-Hispanic Black	460 (7)	244 (3.6)	704 (10.6)	
Hispanic	477 (10.1)	262 (5.1)	739 (15.2)	
Non-Hispanic Asian	177 (3.5)	78 (2)	255 (5.5)	
Non-Hispanic other	95 (3.6)	62 (1.7)	157 (5.3)	
Health insurance				.41
Health insurance	3150 (61.9)	1569 (28.7)	4719 (90.6)	
No health insurance	245 (6.1)	145 (3.3)	390 (9.4)	
Household income (US $)				<.001
<$20,000	432 (8.4)	329 (6)	761 (14.4)	
$20,000‐34,999	322 (5.6)	234 (3.5)	556 (9.1)	
$35,000‐49,999	455 (7.4)	254 (4.2)	709 (11.6)	
$50,000‐74,999	559 (11.5)	290 (5.7)	849 (17.2)	
$75,000‐99,999	469 (9.6)	236 (4.6)	705 (14.2)	
$100,000‐199,999	787 (17.6)	263 (5.3)	1050 (22.9)	
$200,000+	366 (7.7)	103 (2.9)	469 (10.6)	
Geographic region of residence				<.001
Urban or metro	3055 (61.6)	1432 (26.6)	4487 (88.2)	
Rural or nonmetro	340 (6.4)	282 (5.4)	622 (11.8)	

a HINTS 6, question A2d: “Based on the results of your most recent search for information about cancer, how much do you agree or disagree with each of the following statements? The information you found was hard to understand.”

### Cancer Information-Seeking

Table 2 presents the independent association between satisfaction with at-home internet connection and cancer information-seeking experiences in multivariable logistic regression models. Respondents who reported low satisfaction with their home internet connection had higher odds of agreeing that it took a lot of effort to get the cancer information they needed (odds ratio [OR 1.59, 95% CI 1.16‐2.19) and to feel more frustration searching for information (OR 1.46, 95% CI 1.07‐1.98). However, while those with lower satisfaction with their home internet had higher odds of reporting concern about the quality of cancer information they found, this association was not statistically significant (OR 1.37, 95% CI 0.97‐1.92).

**Table 2.** Weighted multivariable logistic regression results regarding cancer information-seeking experiences.

	Effort to get information		Frustration searching for information		Concern about the quality of information	
	Point estimate, odds ratio (95% CI)	*P* value	Point estimate, odds ratio (95% CI)	*P* value	Point estimate, odds ratio (95% CI)	*P* value
Satisfaction with at-home internet						
Reference: high satisfaction with internet	1.0	—^a^	1.0	—	1.0	—
Low satisfaction with internet	1.59 (1.16-2.19)	<.01	1.46 (1.07-1.98)	.02	1.37 (0.97-1.92)	.07
Age (years)						
Reference: 75+	1.0	—	1.0	—	1.0	—
18‐34	0.64 (0.35-1.17)	.14	0.57 (0.32-1.05)	.07	1.74 (1.04-2.90)	.04
35‐49	0.73 (0.46-1.16)	.18	0.73 (0.42-1.27)	.26	1.52 (0.94-2.46)	.09
50‐64	1.16 (0.68-1.99)	.57	0.76 (0.43-1.34)	.34	1.65 (1.02-2.69)	.04
65‐74	0.84 (0.50-1.39)	.48	0.51 (0.29-0.90)	.02	0.99 (0.63-1.58)	.98
Educational attainment						
Reference: postbaccalaureate degree	1.0	—	1.0	—	1.0	—
Less than high school	0.72 (0.25-2.11)	.54	0.53 (0.24-1.18)	.12	0.57 (0.17-1.92)	.36
High school graduate	0.79 (0.44-1.43)	.43	0.91 (0.55-1.49)	.69	0.56 (0.31-0.99)	.04
Some college	0.86 (0.62-1.19)	.35	0.88 (0.56-1.40)	.59	0.66 (0.44-0.99)	.05
Bachelor’s degree	0.88 (0.61-1.26)	.46	0.84 (0.55-1.27)	.39	0.67 (0.48-0.95)	.03
Understanding cancer information						
Reference: easy to understand	1.0	—	1.0	—	1.0	—
Hard to understand	7.67 (5.42-10.86)	<.001	8.53 (6.10-11.94)	<.001	10.28 (7.41-14.27)	<.001
Race and ethnicity						
Reference: non-Hispanic White	1.0	—	1.0	—	—	—
Non-Hispanic Black	1.06 (0.66-1.71)	.81	0.84 (0.46-1.54)	.58	0.76 (0.45-1.28)	.29
Hispanic	1.08 (0.64-1.81)	.77	1.53 (0.78-2.99)	.21	0.96 (0.55-1.67)	.89
Non-Hispanic Asian	1.72 (0.76-3.91)	.19	1.33 (0.53-3.34)	.54	1.35 (0.65-2.78)	.41
Non-Hispanic other	1.61 (0.52-5.00)	.40	2.80 (1.23-6.36)	.01	1.93 (0.80-4.67)	.14
Health insurance						
Reference: health insurance	1.0	—	—	—	—	—
No health insurance	0.81 (0.36-1.78)	.59	0.66 (0.34-1.26)	.20	1.25 (0.58-2.67)	.57
Household income (US $)						
Reference: $200,000+	1.0	—	1.0	—	1.0	—
<$20,000	1.05 (0.52-2.15)	.88	0.69 (0.33-1.44)	.32	0.77 (0.36-1.67)	.50
$20,000‐34,999	0.84 (0.44-1.64)	.61	0.72 (0.37-1.41)	.33	1.25 (0.69-2.28)	.45
$35,000‐49,999	0.73 (0.34-1.58)	.42	0.53 (0.27-1.02)	.06	0.58 (0.33-1.03)	.06
$50,000‐74,999	0.91 (0.51-1.61)	.74	0.49 (0.28-0.87)	.02	0.77 (0.47-1.24)	.27
$75,000‐99,999	0.63 (0.38-1.06)	.08	0.57 (0.30-1.09)	.09	0.94 (0.53-1.69)	.84
$100,000‐199,999	0.92 (0.57-1.48)	.72	0.82 (0.47-1.44)	.49	0.87 (0.54-1.40)	.56
Geographic region of residence						
Reference: urban or metro	1.0	—	1.0	—	1.0	—
Rural or nonmetro	0.80 (0.49-1.32)	.38	1.34 (0.81-2.22)	.26	1.03 (0.60-1.78)	.90

a Not available.

Some sociodemographic characteristics were also independently associated with satisfaction with information seeking. Respondents who reported greater difficulty understanding cancer information had significantly higher odds of agreeing that it took a lot of effort to get the cancer information they needed (OR 7.67, 95% CI 5.42‐10.86), higher odds of reporting frustration searching for information (OR 8.53, 95% CI 6.10‐11.94) and increased odds to be concerned about the quality of health information (OR 10.28, 95% CI 7.41‐14.27). Regarding frustration in searching for information, respondents 65 to 75 had lower odds of reporting frustration searching the cancer information that they needed, compared with respondents 75 years and older (OR 0.57, 95% CI 0.32‐1.05), as well as individuals reporting a household income between US $50,000 and US $74,999 compared with households with a US $200,000 income or higher (OR 0.49, 95% CI 0.28‐0.87). Furthermore, those categorized as non-Hispanic other had higher odds of reporting frustration searching information compared with non-Hispanic Whites (OR 2.80, 95% CI 1.23‐6.36).

For concerns on the quality of health information, respondents aged 18‐34 years old had higher odds of reporting concern about the quality of information found (OR 1.74, 95% CI 1.04‐2.90) compared with respondents aged 75 years and older. Also, those with a high school diploma or GED (OR 0.56, 95% CI 0.31‐0.99), some college (OR 0.66, 95% CI 0.44‐0.99), or a college diploma (OR 0.67, 95% CI 0.48‐0.95) reported lower odds of being concerned about the quality of information compared with respondents with postgraduate education.

### Use of Health Technology

Table 3 includes findings on health information technology use and satisfaction with at-home internet connection among HINTS 6 respondents. Participants who reported lower satisfaction with their home internet had lower odds of reporting accessing their electronic medical portal at least once over the past 12 months (OR 0.54, 95% CI 0.33‐0.89). The same outcome was seen with educational attainment, where compared with those with a postbaccalaureate degree, individuals without a high school diploma (OR 0.16, 95% CI 0.03‐0.79) and those with some college education but no diploma (OR 0.56, 95% CI 0.32‐0.98) had significantly lower odds of reporting access to their electronic medical records at least once over the past 12 months.

**Table 3.** Weighted multivariable logistic regression of satisfaction with internet on telehealth and patient portal use.

	Accessing medical record/patient portal		Receive health care using telehealth	
	Point estimate, odds ratio (95% CI)	*P* value	Point estimate, odds ratio (95% CI)	*P* value
Satisfaction with at-home internet				
Reference: high satisfaction with internet	1.0	—^a^	1.0	—
Low satisfaction with internet	0.54 (0.33-0.89)	.02	0.73 (0.51-1.04)	.08
Age (years)				
Reference: 75+	1.0	—	1.0	—
18‐34	1.00 (0.46-2.17)	.99	1.40 (0.84-2.32)	.19
35‐49	0.83 (0.38-1.82)	.64	1.92 (1.18-3.12)	.01
50‐64	1.35 (0.70-2.63)	.37	1.44 (0.88-2.34)	.14
65‐74	0.87 (0.43-1.74)	.68	1.06 (0.69-1.62)	.80
Educational attainment				
Reference: postbaccalaureate degree	1.0	—	1.0	—
Less than high school	0.16 (0.03-0.79)	.03	0.69 (0.21-2.19)	.52
High school graduate	0.53 (0.27-1.04)	.06	0.65 (0.39-1.09)	.10
Some college	0.56 (0.32-0.98)	.04	0.93 (0.64-1.35)	.68
Bachelor’s degree	0.75 (0.48-1.15)	.18	0.77 (0.54-1.10)	.15
Understanding cancer information				
Reference: easy to understand	1.0	—	1.0	—
Hard to understand	0.72 (0.47-1.12)	.15	0.89 (0.63-1.26)	.52
Race and ethnicity				
Reference: non-Hispanic White	1.0	—	1.0	—
Non-Hispanic Black	0.69 (0.30-1.58)	.37	0.79 (0.38-1.61)	.51
Hispanic	0.62 (0.32-1.19)	.15	1.44 (0.92-2.25)	.11
Non-Hispanic Asian	0.75 (0.13-4.24)	.74	1.01 (0.51-2.01)	.97
Non-Hispanic other	1.33 (0.32-5.62)	.69	1.22 (0.55-2.69)	.62
Health insurance				
Reference: health insurance	1.0	—	1.0	—
No health insurance	0.97 (0.37-2.59)	.95	0.39 (0.19-0.81)	.01
Annual household income				
Reference: $200,000+	1.0	—	1.0	—
<$20,000	1.07 (0.37-3.13)	.90	1.85 (0.96-3.58)	.07
$20,000‐34,999	0.77 (0.26-2.27)	.63	0.93 (0.44-1.99)	.85
$35,000‐49,999	0.73 (0.27-2.00)	.53	0.88 (0.48-1.61)	.66
$50,000‐74,999	0.85 (0.31-2.30)	.74	1.03 (0.66-1.63)	.89
$75,000‐99,999	0.92 (0.37-2.34)	.86	1.27 (0.78-2.07)	.32
$100,000‐199,999	1.20 (0.47-3.11)	.70	1.13 (0.70-1.81)	.61
Geographic region of residence				
Reference: urban or metro	1.0	—	1.0	—
Rural or nonmetro	0.81 (0.43-1.52)	.51	1.01	.98

a Not available.

For the outcome of receiving care through telehealth, while those reporting low satisfaction with their home internet connection had lower odds of receiving care from a health care provider using telehealth over the past 12 months, this association was not statistically significant (OR 0.73, 95% CI 0.51‐1.04). Adults 35 to 49 years had significantly higher odds of receiving care through telehealth compared with respondents 75 years and older (OR 1.92, 95% CI 1.18‐3.12). Finally, respondents without health insurance had lower odds of receiving care from a health care provider using telehealth over the past 12 months, compared with respondents with insurance (OR 0.39, 95% CI 0.19‐0.81).

## Discussion

### Principal Findings

The purpose of this study was to assess how satisfaction with at-home internet to meet health needs was associated with cancer health information-seeking perceptions, digital health technology use, and respondent characteristics in a population-level, nationally representative sample of US adults. Our findings pinpoint disparities in the level of satisfaction with internet connection that Americans report by age, level of education, income, and geography. Our assessment of perceived satisfaction with at-home internet connection to meet health-related needs uses a novel item, and the results are consistent with previous studies that examined other predictors of internet access and use. A previous study using data from 8 HINTS iterations (2003‐2017) found age to be significantly associated with overall internet use, broadband internet access, and internet use through a cellular network, with younger respondents (18-34 years old) reporting greater access than older age groups [28]. Previous studies have found that use of broadband internet is associated with higher levels of educational attainment and higher household income. Our analysis suggests that more satisfaction with home internet services to meet health needs is an important predictor of information-seeking experiences and use of some digital health technology tools [29-32].

### Satisfaction With Internet and Cancer Information-Seeking

In our analysis, negative experiences with health information-seeking, particularly increased effort and frustration when searching cancer information, were significantly associated with lower at-home internet satisfaction. These findings provide evidence of the relationship between internet quality and health-information seeking. Previous work on health-information seeking perceptions has assessed effort, frustration, quality of the resources, and challenges to understand health information within the context of different types of literacy, including health and e-Health literacy [33,34]. Furthermore, greater frustration in information-seeking efforts can hinder use of informational resources provided through trusted sources, such as health care providers [35]. Such results suggest that the intersection between limited or unsatisfactory access to quality internet and low literacy should be assessed together to determine their potential as a deterrent to health information-seeking.

### Satisfaction With Internet and Health Technology Use

Our findings suggest that individuals who report lower satisfaction with their home internet connection have significantly lower odds of using certain internet-supported health technologies, particularly accessing electronic health records. Such results align with previous assessments of digital health technology use. While use of electronic health records is widely promoted as a complementary resource to in-person care, our findings suggest that lack of health insurance is a persistent barrier for their uptake. A study using previous cycles of HINTS data (2017‐2018) found an association between having health insurance and use of patient portals; this suggests that while the COVID-19 pandemic might have amplified the use of some internet health tools, health insurance access might be a persistent barrier toward uptake of some digital health technology [36].

Although our assessment on the impact of satisfaction with at-home internet and telehealth use was not statistically significant, older age and lack of health insurance were shown as meaningful predictors of lower telehealth use, paralleling some recent literature. A 2021 cross-sectional study on the use of telehealth and telepharmacy services in US adults found respondents over 55 were significantly less willing to use telehealth compared with younger respondents [37]. A 2024 scoping review on global access and payment to telehealth found that in the United States, where health care is primarily covered by private insurers, lack of insurance presents an ongoing challenge toward telehealth coverage [38]. These findings highlight the challenges of sustained adoption of digital health tools by certain demographic groups, and it behooves us to understand how personal preferences, such as interpersonal interactions with providers, and policy-level challenges in health care coverage might affect the benefits of telehealth services.

The COVID-19 pandemic underscored the value of digital health technologies in providing and sustaining care when in-person health care was not feasible, and the potential toward expedient adoption of these tools under these types of crises [39,40]. However, it also brought attention to pervasive “intervention-generated inequalities” in digital health, where patients who have the advantages of infrastructural (ie, higher quality internet) and financial access to technology benefit, but those without do not [31]. The importance of high-quality access is illustrated through the Federal government’s Healthy People initiative, which outlines major national objectives and goals toward improving health promotion and the prevention of disease and includes an objective to “increase the proportion of adults with broadband Internet.” [41]. While the Federal Communications Commission (FCC) received funding to increase broadband coverage during the COVID-19 pandemic, sustainability of quality internet in the long term will play a pivotal role for the dissemination of digital health resources, particularly among poor, underserved, or rural communities [42,43].

### Strengths and Limitations

Our study has notable strengths and some limitations. HINTS 6 incorporates a probability-based survey with a relatively large, representative sample of US adults and is one of the first federal, public-use surveys to include an item about satisfaction with at-home internet. Furthermore, the inclusion of the final sample weight and replicate weights allowed us to calculate population-level point estimates and accurate standard errors, respectively.

Nevertheless, our analysis presents some limitations. First, our findings are reflective of members of the US population who have access and use the internet; while approximately 96% of US adults report using the internet, it might exclude individuals with no access to internet services [44]. Second, in the analysis using telehealth access as an outcome variable, we did not differentiate between respondents who answered “No” due to not needing an appointment with a provider as opposed to not having satisfactory access to the internet, though we did exclude those who didn’t see their provider over the past year. Third, in this study, we used a proxy item (“Based on the results of your most recent search for information about cancer, how much do you agree or disagree with each of the following statements? The information you found was hard to understand”) to assess health literacy, which is not an exhaustive measure of this factor. Finally, HINTS is a cross-sectional survey, so temporality among associations cannot be established.

### Conclusions

This study suggests that lower self-reported satisfaction with home internet connection can have negative impacts on perceived health information-seeking experiences and use of some digital health resources. As digital health tools become more ubiquitous in health care, efforts to enhance access to high-speed, quality internet, particularly among underserved, hard-to-reach rural communities, will be needed to lessen the digital gap. Future research should also continue to assess satisfaction with at-home internet connection, along with broadband access metrics, to better examine the relationship between user perceptions about internet access and resultant outcomes.

## Supplementary material

10.2196/69606 Multimedia Appendix 1 Health Information National Trends Survey (HINTS) 6 sample description using the Preferred Reporting Items for Complex Sample Survey Analysis.

## Abbreviations

EHR: electronic health record
HINTS: Health Information National Trends Survey
IRB: institutional review board
OR: odds ratio
