# Supplementary material for: Satisfaction With Internet Access, Cancer Information-Seeking, and Digital Health Technology: Cross-Sectional Survey Assessment
Source: J Med Internet Res. 2025 Aug 27;27:e69606. doi: 10.2196/69606 (PMC12384670; doi:10.2196/69606)
Supplement: Multimedia Appendix 1 [file jmir-v27-e69606-s001.pdf]

Supplementary Table 1. HINTS 6 sample description using the Preferred Reporting Items for Complex Sample Survey Analysis (PRICSSA)

**PRICSSA Item**	**Sample Description**
Name and wave of survey	HINTS 6 (2022)
Data collection mode	Mail/Web (self-administered)
Dates of data collection	03/07/2022-11/08/2022
Target population	Civilian, non-institutionalized adults aged 18 or older living in the United States
Populations excluded	Persons living in an institutionalized setting (i.e. nursing homes, prisons, military bases, college dormitories)
Design	Two-stage, stratified sample design: first, a stratified sample of addresses was selected from a file of residential addresses; second, one adult was selected within each sampled household. HINTS 6 sample design expands two-sampling strata of high minority and low minority into four by further breaking them out by rural and urban geographic areas.
Variance estimation	'Delete one' jackknife (JK1) replication method
Weight and design variables	
Weight	person_finwt0
Replication method	person_finwt1-person_finwt50
Unweighted total sample size:	6,252
Weighted total sample size:	258,418,467
Response rate:	25.90%
Location of example code:	See "Overview of the HINTS 6 Survey (2022) and Data Analysis Recommendations" (SAS/SPSS/STATA/R versions available)
